# Seasonal variation in *Aspergillus* abundance in captive penguin burrow sands and its implication for aspergillosis risk in Japan

**DOI:** 10.3389/fvets.2025.1708049

**Published:** 2026-01-13

**Authors:** Shiori Takanobu, Yuri Araki, Rie Nitta, Hideaki Shindo, Naoya Matsumoto, Megumi Itoh, Kazutaka Yamada, Takahito Toyotome

**Affiliations:** 1 Department of Veterinary Medicine, Obihiro University of Agriculture and Veterinary Medicine, Obihiro, Hokkaido, Japan; 2 Shimonoseki Marine Science Museum, Shimonoseki, Yamaguchi, Japan; 3 Azabu University, Sagamihara, Kanagawa, Japan; 4 Department of Pharmaceutical Sciences, School of Pharmacy at Narita, International University of Health and Welfare, Narita, Chiba, Japan; 5 Medical Mycology Research Center, Chiba University, Chiba, Japan

**Keywords:** aspergillosis risk, *Aspergillus*, burrow sand, penguin, seasonal variation

## Abstract

**Background:**

Aspergillosis is a major fungal disease in penguins. However, seasonal variation of *Aspergillus* spp. in burrow sands and its association with meteorological factors remain poorly characterized, particularly under Japan’s climatic conditions.

**Objectives:**

This study aimed to examine seasonal changes in *Aspergillus* spp. abundance in Humboldt penguin burrows and test whether temperature and other environmental factors correlated with fungal positivity.

**Methods:**

From June 2023 to October 2024, 158 sand samples were collected from burrows and surrounding areas at an outdoor Humboldt penguin (*Spheniscus humboldti*) facility in Shimonoseki, Japan. Fungal colonies were cultured and identified morphologically and by sequencing.

**Results:**

*Aspergillus* spp. positivity peaked from July to October in both years. Average temperature showed strong positive correlation with fungal positivity (*r* = 0.781, *p* < 0.01), while other meteorological factors exhibited weaker associations. Multiple *Aspergillus* spp. were identified, including known pathogenic *Aspergillus* sections *Fumigati*, *Nigri*, *Flavi*, and *Terrei*.

**Conclusion:**

Temperature demonstrated the strongest correlation with *Aspergillus* spp. positivity, suggesting seasonal monitoring is critical for managing aspergillosis risk in captive penguins. These findings provide a basis for future multi-site studies to improve fungal disease prevention strategies.

## Introduction

Aspergillosis is a common and often fatal fungal disease in birds, caused predominantly by *Aspergillus fumigatus* and other species of the genus *Aspergillus* (1, 2). Although the exact mechanism is unclear, several anatomical characteristics of avian species—including the absence of an epiglottis, the absence of a diaphragm, and the limited distribution of pseudostratified ciliated columnar cells throughout the respiratory tract—might contribute to the higher susceptibility of avian species to aspergillosis (3). Among avian species, penguins are particularly susceptible to aspergillosis due to the stressful conditions often encountered in both captive and wild environments and many cases have been reported in captive and free-ranging penguins (4–9). In addition, penguin species, especially Antarctic and sub-Antarctic penguin species, are susceptible to heat stress, which is considered a factor that may increase the risk of aspergillosis as a secondary infection (10).

Penguins spend long periods of time in their burrows, especially during the breeding season. For example, the incubation period for Humboldt penguins (*Spheniscus humboldti*) is approximately 40 days (11). In the wild, Humboldt penguins dig burrows in guano layers or burrow in spaces between rocks (12). Because temperate penguin species including Humboldt penguins are heat-tolerant, they kept in outdoor facilities with vegetation that were the focus of this study dig burrows in the ground and line them with plant material and other nesting materials, such as stones (10). The enclosed nature of these burrows, combined with prolonged contact with moist organic substrates, may create microenvironments favorable for fungal proliferation, including *Aspergillus* spp.

Due to its location in the temperate monsoon zone, Japan exhibits significant seasonal variability, with high temperatures and humidity in summer and cold, dry conditions in winter. *Aspergillus* spp., particularly *A. fumigatus* and *A. flavus*, are known for their thermotolerance compared to other environmental fungi (13, 14). *Aspergillus* spp., including *A. fumigatus*, exhibit remarkable thermotolerance and can germinate at temperatures as high as 40 °C (14). Under Japan’s summer conditions, this characteristic is consistent with the possibility that *Aspergillus* spp. become more prevalent under high-temperature conditions and potential exposure risk for penguins.

Despite the potential importance of seasonal dynamics, limited information is available regarding the temporal dynamics of *Aspergillus* spp. abundance in these burrow sands and the seasonal factors influencing their growth. Understanding these dynamics is essential for identifying periods of heightened disease risk and implementing targeted conservation and veterinary interventions.

In this study, we conducted a quantitative analysis of *Aspergillus* spp. abundance in the sands of penguin burrows over multiple seasons. By correlating fungal load with environmental variables, we aimed to identify the period(s) of greatest aspergillosis risk for penguins and provide evidence-based recommendations for disease mitigation efforts. To our knowledge, this is the first longitudinal study in Japan to investigate seasonal *Aspergillus* spp. dynamics in penguin burrow sands.

## Materials and methods

### Ethical considerations regarding the use of animals

All samples were collected in accordance with the standard husbandry and hygiene guidelines of the zoo and no invasive procedures were performed.

### Description of the penguin population

The sand sampling from penguin burrows was performed in Shimonoseki Marine Science Museum (33° 57′16.1″N, 130° 56′32.7″E) from April 2023 to October 2024. Preliminary sampling was performed in April and May 2023. The aquarium has a colony of Humboldt penguins (*Spheniscus humboldti*). In June 2023, 43 Humboldt penguins were kept. The colony’s enclosure is located outdoors, with a total area of 751.43 m^2^, of which 100 m^2^ is land and 45 m^2^ is a pool. Demographic information of the colony was shown in Supplementary Table 1. Two Humboldt penguins (seven- and nineteen-year-old female penguins) in the colony were suspected to having suspected aspergillosis between April 2023 and October 2024. Nebulizer therapy to treat an individual was given in a burrow (Burrow ID 11) daily from October 11, 2023, to July 30, 2024, and thereafter only on the following dates: September 2, 4, 5, 7, 9, 11, 13, 16, 18, 20, 23, 25, 27, and 30, 2024; October 3, 7, 10, 13, 16, 18, 21, 23, 25, and 27, 2024. Both individuals recovered during the study period. One Humboldt penguin (fourteen-year-old female penguin) was confirmed to have aspergillosis. However, because the individual was in a different location at the time of initial onset, it is considered that the infection did not occur within sampling area targeted in this study. No new cases of aspergillosis occurred within the facility in 2024.

### Sampling

Preliminary sampling was performed at four burrows, burrow ID 10, 11, 15, and 17. From June 2023 to October 2024, sand samples were collected from a total of nine locations, comprising seven burrow sites and two non-burrow-associated sites. The latter included one sample obtained from beneath a plant and another from an area adjacent to cleaning supplies. All burrows, except burrow ID 17, remained accessible to penguins throughout the study period. Burrow ID 17 was rendered unavailable after December 22, 2023. These sampling burrows and points, except burrow ID 17, were frequently flooded during and after rainfall. The sampling points are detailed in Supplementary Figures 1, 2. Sand samples used in this study were collected from the surface layer of the sampling points during routine cleaning procedures. Cleaning was performed approximately every two weeks. Samples were collected approximately once a month, with the detailed sampling schedule outlined in Supplementary Table 2. Cleaning involved removing surface sand contaminated primarily with feces, followed by disinfection using a commercially available detergent containing soy fatty acids until October 20, 2023. After that date, a chlorine solution (0.012% sodium hypochlorite solution) was applied using a spray bottle, and fresh sand was subsequently added. Each topsoil (including 1–2 cm of sand) sample was collected with gloves or plastic bags, and then they were transferred into sterile 50-mL centrifuge tubes for storage. A total of 158 sand samples were collected. Those samples were stored until analysis at 4 °C.

### Culture of fungal species from sand samples

Sand samples were weighed about one gram and subsequently suspended in 0.05% Tween 20 at a volume equivalent to nine times its mass. After vigorous mixing for 10 s, 3 mL suspension and 12 mL 1/2 dichloran glycerol 18 (DG18) agar (Merck, Darmstadt, Germany). The 1/2 DG18 media was prepared at half-strength according to manufacturer’s instructions, while the volume of glycerol added remained unchanged from the manufacturer’s instruction. Each 5 mL suspension was overlaid on three DG18 agar plates, and those were incubated at 35 °C by two weeks. The presence of *Aspergillus* spp. colonies on the plates were confirmed by visual inspection and observation under a microscope. Colonies that were visually and microscopically identified as *Aspergillus* spp. were counted, subsequently isolated, and subjected to further identification procedures. The monthly *Aspergillus* positivity rate was calculated as the proportion of sand samples collected in each month that yielded at least one colony morphologically identified as *Aspergillus* spp.

### Fungal genomic DNA preparation, amplification of targeted region, and sequencing

To prepare genomic DNA from fungal isolates, mycelia cultured in potato dextrose broth (Becton, Dickinson and Company, Franklin Lakes, NJ, United States) with 0.1% yeast extract (Oriental Yeast Co., Ltd., Tokyo, Japan) were collected and homogenized with 1.0 mm-diameter zirconia beads (ZB-10, TOMY SEIKO Co., Ltd., Tokyo, Japan). DNA extraction with phenol:chloroform:isoamyl alcohol was performed using the procedure described previously (15). DNA was used for subsequent PCR amplification and sequencing internal transcribed spacer (ITS) regions and partial *β*-tubulin and calmodulin genes. For ITS, β-tubulin, and calmodulin regions, the primer pairs of ITS4 and ITS5, Bt2a and Bt2b, and cmd5 and cmd6 were used, respectively. If it was thought to be *Aspergillus* section *Nigri*, the primer pairs of AnBt2a and AnBt2b and AnigCMF and AnigCMR, instead of Bt2a/Bt2b and Cmd5/Cmd6 pairs, were used for the amplifications of partial *β*-tubulin and calmodulin genes, respectively. These primer sequences were described in a previous report (16). Emeraldamp PCR Master Mix (Takara Bio Inc., Shiga, Japan) was used as the reagent for 10-μL reaction and PCR was performed using a GeneAmp PCR System 2,700 thermal cycler (Applied Biosystems, Waltham, MA, United States). PCR amplification was performed for 40 cycles consisting of denaturation at 98 °C for 10 s, annealing at 52 °C for 30 s, and extension at 72 °C for 60 s. Sanger sequencing was carried out on a 3,500 Genetic Analyzer (Applied Biosystems, Waltham, MA, United States) according to the manufacturer’s instructions. The obtained sequences were analyzed by BLASTN.^1^

### Meteorological data

Meteorological data for Shimonoseki city were obtained from the historical records provided by the Japan Meteorological Agency (JMA).

### Statistical analysis

Pearson’s correlation coefficients (*r*) were calculated to assess the relationships between monthly *Aspergillus* positivity rates and various meteorological parameters, including average temperature, precipitation, humidity, and sunshine duration. The sample size for the correlation analysis was *n* = 16 (monthly data points from June 2023 to October 2024). No multivariate adjustment was performed; correlations were assessed individually for each parameter. These calculations were performed using the CORREL function in Microsoft Excel (Microsoft Corporation, Redmond, WA, United States), and *p*-values were calculated based on the t-distribution formula (df = 14). Statistical significance was interpreted at *p* < 0.01.

## Results and discussion

Preliminary sampling conducted in April and May 2023 revealed the presence of only two *Aspergillus* colonies in sand samples collected from burrow IDs 10 and 11 (Supplementary Figure 3). These colonies were identified as *Aspergillus cejipii* and *A. fumigatus*.

**Figure 1:**
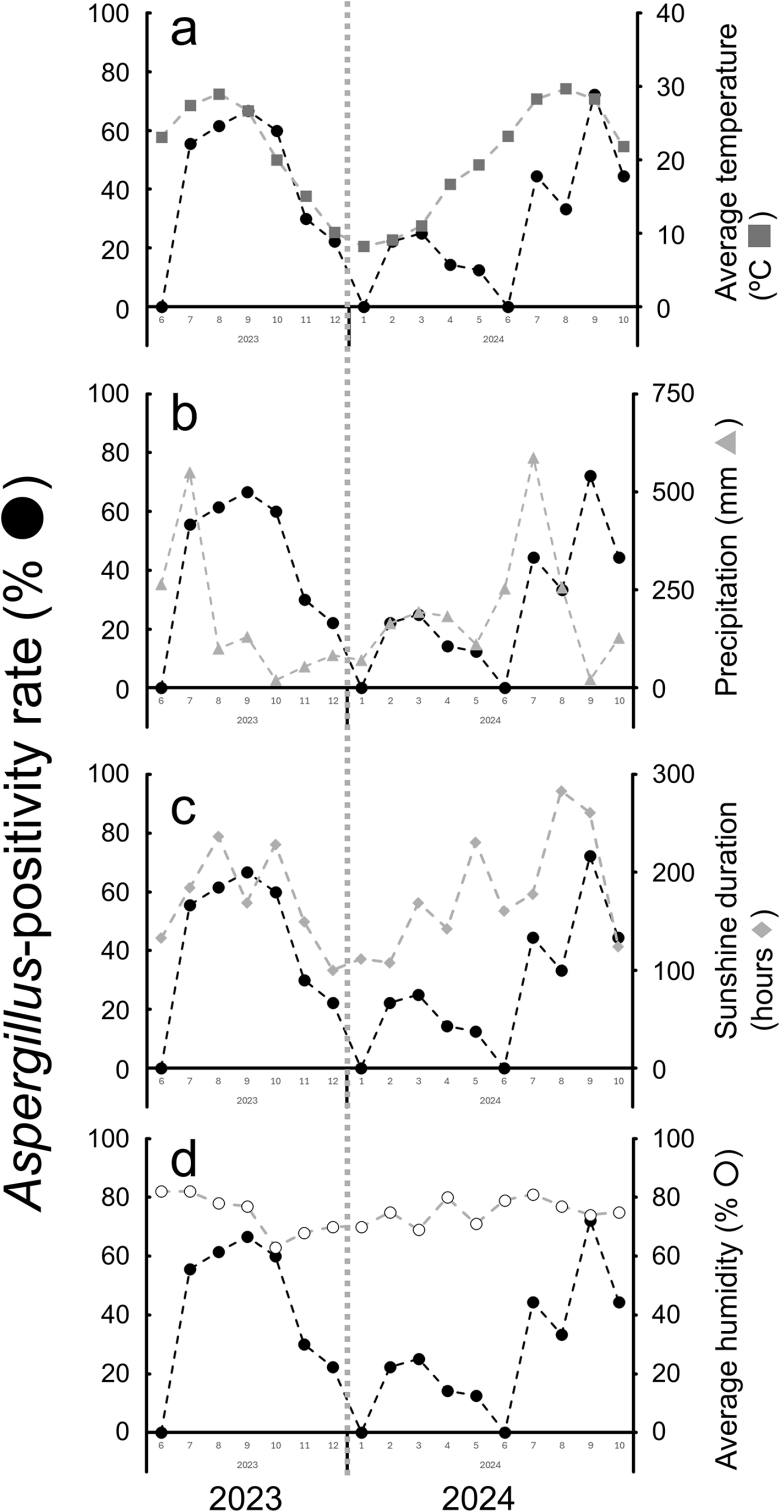
Monthly variation in Aspergillus positivity rates and meteorological parameters in Shimonoseki, Japan, from June 2023 to October 2024. **(a)** Aspergillus positivity rates (%, 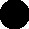) in sand samples from Humboldt penguin burrows plotted against average temperature (°C, 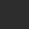). **(b)** Aspergillus positivity rates (%, 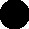) and monthly precipitation (mm, 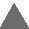). **(c)** Aspergillus positivity rates (%, 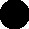) and sunshine duration (hours, 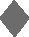). **(d)** Aspergillus positivity rates (%, 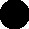) and average humidity (%, 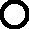).

From June 2023 to October 2024, a total of 158 sand samples were collected (Supplementary Datasheet 1), among which 60 samples were confirmed as *Aspergillus* spp.-positive samples (Table 1; Supplementary Datasheet 1). The relationship between *Aspergillus* spp. positivity rates and various meteorological parameters is shown in Figure 1 and Table 1. A wide fluctuation in the monthly *Aspergillus* spp. positivity rate was observed during the study period, with values spanning from 0 to 73%. Elevated *Aspergillus* spp. positivity rates were consistently observed during the months of July to October in both 2023 and 2024 (Figure 1). Notably, the increase in monthly average temperature preceded the rise in *Aspergillus* spp. positivity rates by approximately one month. The statistical analysis revealed a strong positive correlation between temperature and positivity rate, with a coefficient of correlation (*r*) of 0.781 (*p* = 0.00035). In contrast, no strong correlations were observed between *Aspergillus* spp. positivity rates and other meteorological parameters such as monthly precipitation (0.316, *p* = 0.23), monthly sunshine duration (0.457, *p* = 0.074), or monthly average humidity (0.556, *p* = 0.025). These findings suggest that rising temperatures may be associated with increased *Aspergillus* spp. proliferation, considering the temporal precedence and moderate correlation with positivity rates. Our data align with a previous report that warmer outdoor temperatures increase airborne *Aspergillus* loads in penguin habitat (17). The strong correlation between temperature and fungal positivity, combined with the thermotolerance of *Aspergillus* spp., suggests that elevated summer temperatures appear to be associated with the ecological prevalence of *Aspergillus* spp., explaining the observed data. Such ecological dominance under elevated temperatures may partly explain the seasonal peaks in *Aspergillus* spp. positivity observed in this study. Temperature and humidity are interrelated, and both are considered to influence the growth of *Aspergillus* spp. In our analysis, humidity tended to fluctuate in correlation with temperature, but with a lead time of approximately one to two months. However, no strong correlation was observed between the humidity level and the positivity rate for *Aspergillus* spp. in this study. Further investigation may be warranted to clarify the potential association between humidity and the Aspergillus positivity rate. Furthermore, in both 2023 and 2024, the highest monthly precipitation was recorded in July, coinciding with the rainy season in Japan. Although precipitation alone was not strongly correlated with *Aspergillus* spp. positivity rates, its seasonal peak alongside rising temperatures could indicate a synergistic effect, where increased moisture and warmth together create favorable conditions for fungal growth. The microclimatic conditions within Humboldt penguin burrows may further enhance the suitability for *Aspergillus* spp. growth. On the other hand, these burrows may serve to prevent excessive heat accumulation caused by solar radiation by providing protection from direct sunlight. Our preliminary measurements showed that, during peak summer (July 30, 2024), soil temperature adjacent to burrows (burrow IDs 11 and 12) reached around 45 °C, whereas burrow interiors remained around 29.5 °C, a difference of more than 15 °C. Richards et al. reported that burrows stabilize seabird nest temperatures (18). Such buffering may protect birds from extreme conditions but potentially favor *Aspergillus* persistence when organic matter is present. They demonstrated that burrows stabilize nest temperatures and protect seabird chicks. Such warm conditions may amplify the competitive advantage of *Aspergillus* spp. over other sand microorganisms, particularly during the summer months.

**Table 1:** Monthly *Aspergillus* positivity rates in burrow sand samples and corresponding meteorological data in Shimonoseki, Japan (June 2023–October 2024).

Year	2023							2024									
Month	6	7	8	9	10	11	12	1	2	3	4	5	6	7	8	9	10
*Aspergillus*-positive samples / total samples (% positive)	0/8 (0.0)	5/9 (55.6)	8/13 (61.5)	6/9 (66.7)	6/10 (60.0)	3/10 (30.0)	2/9 (22.2)	0/6 (0.0)	2/9 (22.2)	2/8 (25.0)	1/7 (14.3)	1/8 (12.5)	0/7 (0.0)	4/9 (44.4)	3/9 (33.3)	13/18 (72.2)	4/9 (44.4)
Average temperature (°C)	23.1	27.4	29	26.7	20	15.1	10.1	8.2	9.1	11	16.7	19.3	23.2	28.3	29.7	28.3	21.8
Monthly precipitation (mm)	264.5	549.5	99.5	131	20.5	54.5	83.5	71.5	165	194	182.5	110.5	252.5	587.5	256.5	22	127.5
Monthly sunshine duration (hours)	132.8	184.4	236.6	169	228.4	149.6	99.8	111.6	107.7	168.8	142.5	230.8	160.4	177.8	283.2	261.1	123.8
Average humidity (%)	82	82	78	77	63	68	70	70	75	69	80	71	79	81	77	74	75
Section *Nigri*		+	+	+	+	+	+					+		+	+	+	+
Section *Flavi*				+	+	+				+					+	+	+
Section *Terrei*			+	+	+	+			+		+					+	+
Section *Fumigati*		+	+		+		+		+	+	+			+		+	
Section *Nidulantes*				+	+	+			+						+		
Other section		+^a^		+^b^					+^c^								

^a, b, c,^ Detected species belonging to *Aspergillus* sections *Clavati* (a), *Candidi* (b), and *Aspergillus* (c). Blank cells indicate that no isolates belonging to the respective *Aspergillus* section were detected in sand samples collected during the corresponding month.

As part of the sampling procedure, surface soil in several burrows was replaced as a sanitation measure. Although a direct causal relationship cannot be confirmed, this intervention may have played a role in reducing environmental contamination, as no new cases of aspergillosis were observed in 2024.

No clear differences in trends were observed among the individual burrows. However, in Burrow ID 11, nebulization of liposomal amphotericin B was carried out during the study period because a treated individual was present. Although the Dixon test did not reveal a statistically significant difference, the rate of *Aspergillus* spp. positivity in this burrow was lower than in the other burrows (Supplementary Figure 4). Additionally, two of the three samples that tested positive for *Aspergillus* spp. from Burrow ID 11 were obtained prior to the start of nebulization or during a one-month suspension of the treatment. These observations may indicate an effect of residual amphotericin B following nebulization.

A total of 141 colonies exhibiting distinct morphological characteristics on agar plates were selectively isolated and cultured for subsequent fungal species identification. Details of the *Aspergillus* spp. isolates, including their taxonomic classification at the section and species levels and the corresponding sampling sites, are summarized in Supplementary Table 3. Of the 141 fungal isolates, 11 failed to yield DNA fragments suitable for sequencing due to unknown causes, limiting their identification to the section level. Of the 141 isolates obtained, 35 were identified as the same *Aspergillus* spp. isolated from the same sand samples, based on the identification results. The most frequently detected section was *Aspergillus* section *Nigri* (*n* = 43, 35.0%), followed by *Aspergillus* section *Flavi* (*n* = 31, 25.2%), *Aspergillus* section *Terrei* (*n* = 19, 15.5%), *Aspergillus* section *Fumigati* (*n* = 15, 12.2%), and *Aspergillus* section *Nidulantes* (*n* = 10, 8.1%). A small number of isolates (*n* = 5, 4.1%) were assigned to other sections. *A. fumigatus* belonging to *Aspergillus* section *Fumigati*, which is well-known as a major cause of aspergillosis in avian species (19), ranked as the third most prevalent species (12 isolates were obtained in the research term) among 123 isolates. Some species belonging to *Aspergillus* sections *Nigri*, *Terrei*, and *Flavi*, such as *A. tubingensis*, *A. terreus*, and *A. flavus*, are also known as causative agents of aspergillosis, as well as *A. fumigatus* (19). Sixteen isolates of *A. tubingensis*, a member of the section *Nigri*, were obtained in this study. As reported in several studies (20–22), *A. tubingensis* showed higher minimum inhibitory concentration (MIC) distribution for azole antifungals compared to other representative species within *Aspergillus* section *Nigri*, such as *A. niger* (including its synonym *A. welwitschiae*). Thirteen isolates of *A. pseudonomiae*, a member of the section *Flavi*, were obtained in this study. The species is recognized as an aflatoxin producer, as well as *A. flavus*, the representative species of *Aspergillus* section *Flavi* (9 isolates were identified as *A. flavus* in this study). Recently, an aspergillosis case caused by *A. pseudonomiae* in Okinawa Rail was reported (under review). In addition to *A. flavus*, *A. pseudonomiae* may also need to be considered as a potentially important causative agent of avian aspergillosis. Seven isolates were identified as *A. terreus*, a representative species of *Aspergillus* section *Terrei*. *A. terreus* was also recognized as a causative agent of aspergillosis and case reports in pigeon (23) and Okinawa Rail were found (under review). *A. sydowii*, a representative member of *Aspergillus* section *Nidulantes* (24), was also found as a major isolated species in this study (9 isolates were obtained). Although *A. sydowii* is also recognized as a causative agent of aspergillosis in humans (25), Della Vedova identified the species in a Swinhoe’s pheasant (*Lophura swinhoii*) (1, 26). Although *Aspergillus* sections *Nigri*, *Flavi*, *Terrei*, and *Nidulantes* are not typical causative agents of avian aspergillosis, it is conceivable that under conditions of thermal stress in captive penguins immunosuppression may predispose these birds to infection by such atypical *Aspergillus* spp.

Despite the strengths of this study in elucidating seasonal patterns of *Aspergillus* spp. abundance in penguin burrow sands, several limitations should be acknowledged. First, the study was conducted at a single outdoor facility in Shimonoseki City, Japan, which may limit generalizability to other captive environments with different climatic conditions, husbandry practices, or sand compositions. Multi-site investigations across diverse geographic regions and long-term monitoring are needed to confirm the observed seasonal trends and assess interannual variability, including the influence of atypical climatic events. Second, the relatively short duration of the study (approximately 17 months) allowed observation of two summer seasons but may not fully capture year-to-year fluctuations. Third, methodological constraints included the failure of 11 samples to yield DNA fragments for sequencing and the prolonged storage of samples (4–131 days) prior to processing, which could have affected fungal viability despite no observed correlation with colony counts. Fourth, physical and chemical properties of burrow sands—such as moisture content, organic matter, and nutrient composition—were not analyzed, although these factors are known to influence fungal growth. Additionally, air circulation within burrows was not directly measured; their structure, typically featuring a single entrance and a ventilation opening on the opposite side, suggests that while some airflow occurs, overall air movement remains limited. Our observations indicate that burrow interiors remained cooler than surrounding soil during summer, but the limited number of samples collected from outside burrows restricts conclusions about microenvironmental effects. Fifth, fungal diversity was not assessed using ecological indices such as Shannon or Simpson because the analysis focused exclusively on *Aspergillus* spp. colonies. Finally, reliance on culture-based isolation excluded non-culturable species and may not fully represent the environmental fungal community. Future studies should incorporate substrate profiling, broader culture-based approaches, and high-throughput sequencing to provide a more comprehensive understanding of fungal ecology in penguin habitats.

## Conclusion

This study demonstrates that temperature shows the strongest association with seasonal fluctuations of *Aspergillus* spp. in captive Humboldt penguin burrow sands under Japan’s climatic conditions. The strong temperature–positivity correlation underscores the need for targeted health monitoring and environmental management during the high-risk summer season. These findings provide a practical basis for preventive strategies in zoological facilities and highlight the importance of multi-site and long-term investigations to validate and expand upon these observations.

## Data Availability

The original contributions presented in the study are included in the article/Supplementary material, further inquiries can be directed to the corresponding author.

## Footnotes

1
https://blast.ncbi.nlm.nih.gov/Blast.cgi?PROGRAM=blastn&PAGE_TYPE=BlastSearch&LINK_LOC=blasthome
