# Supplementary figures and images for: Seasonal variation in Aspergillus abundance in captive penguin burrow sands and its implication for aspergillosis risk in Japan
Source: Front Vet Sci. 2026 Jan 13;12:1708049. doi: 10.3389/fvets.2025.1708049 (PMC12836384; doi:10.3389/fvets.2025.1708049)

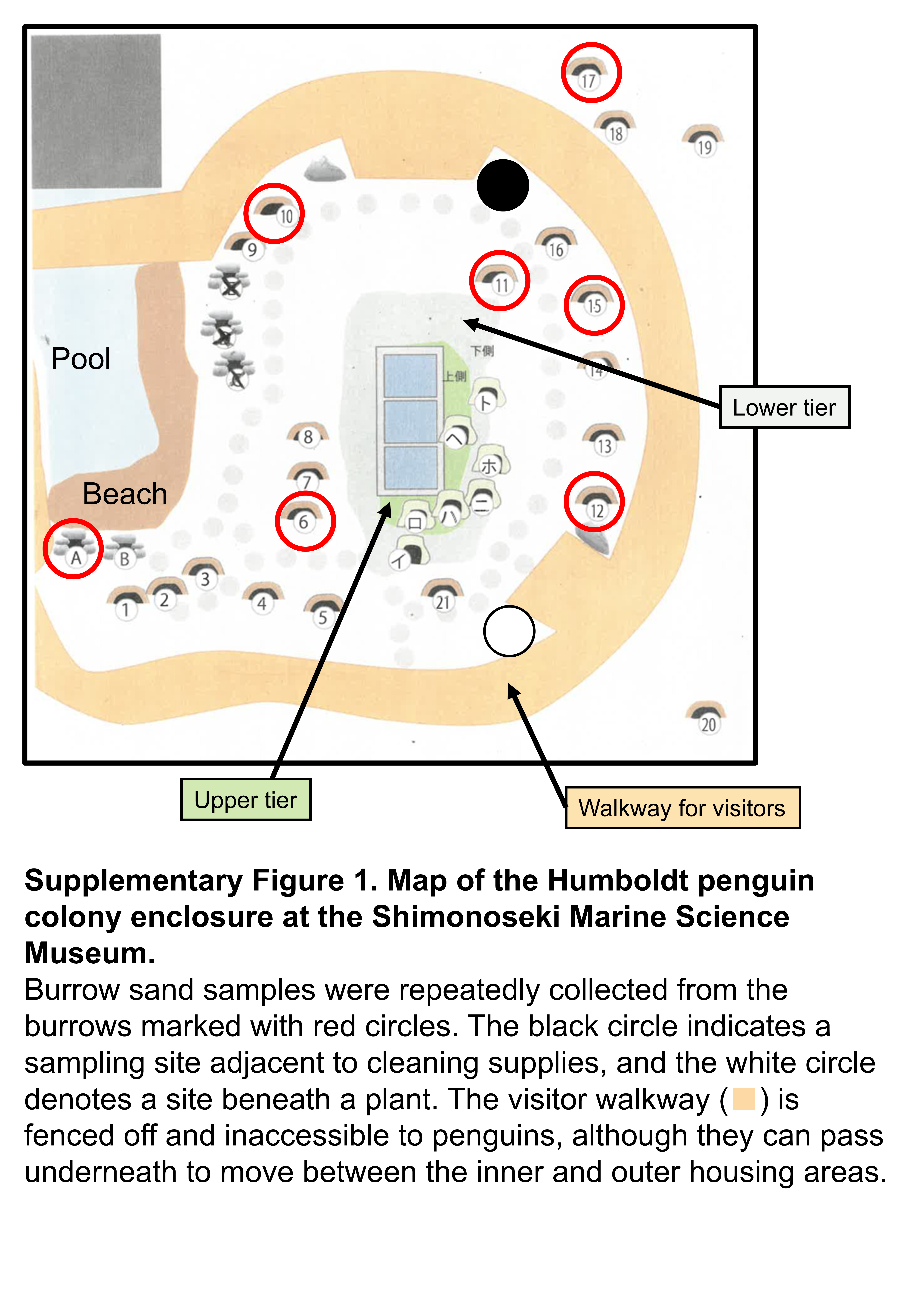

Supplement: Supplementary file 2 [file Image_1.TIF]

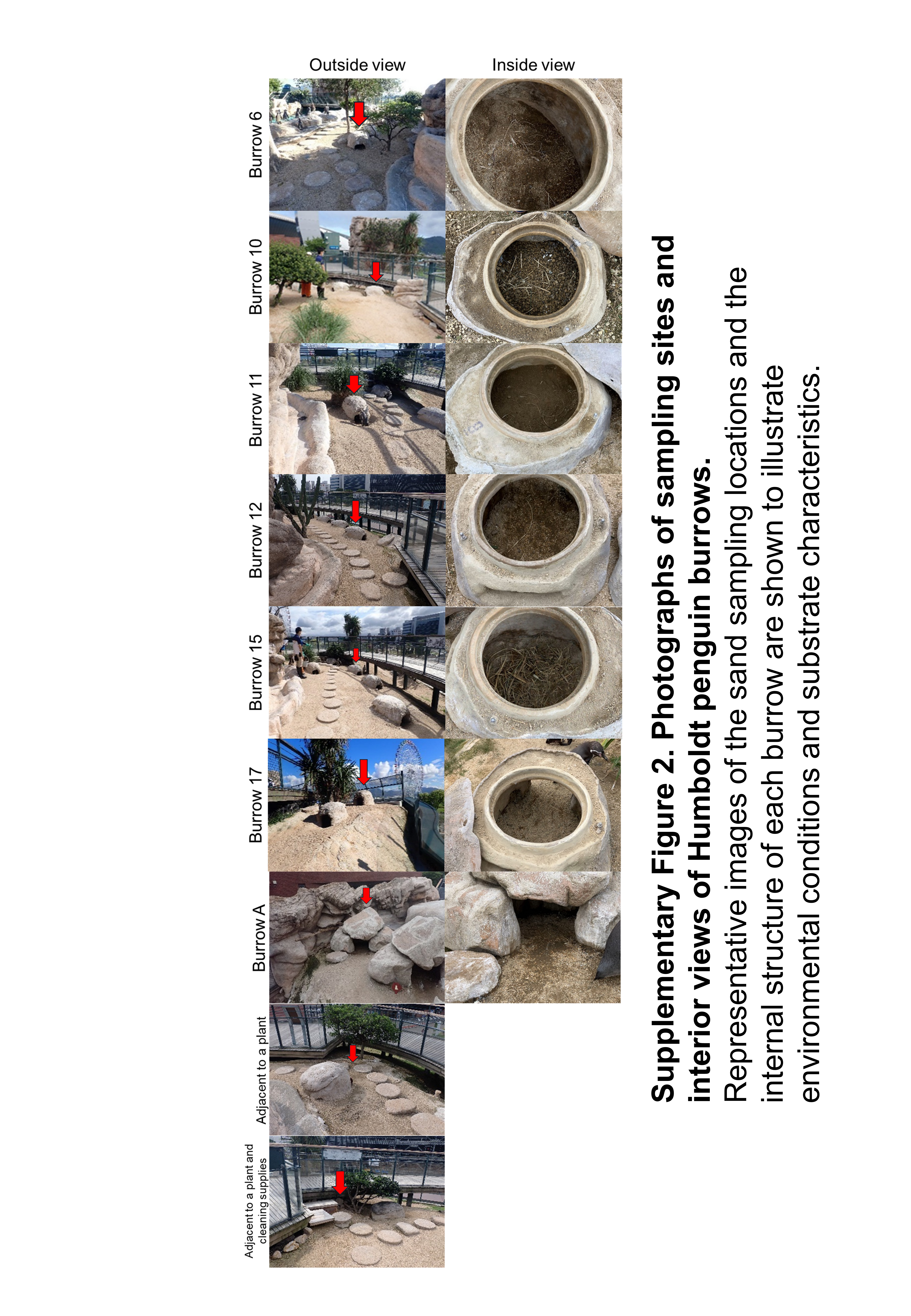

Supplement: Supplementary file 3 [file Image_2.TIF]

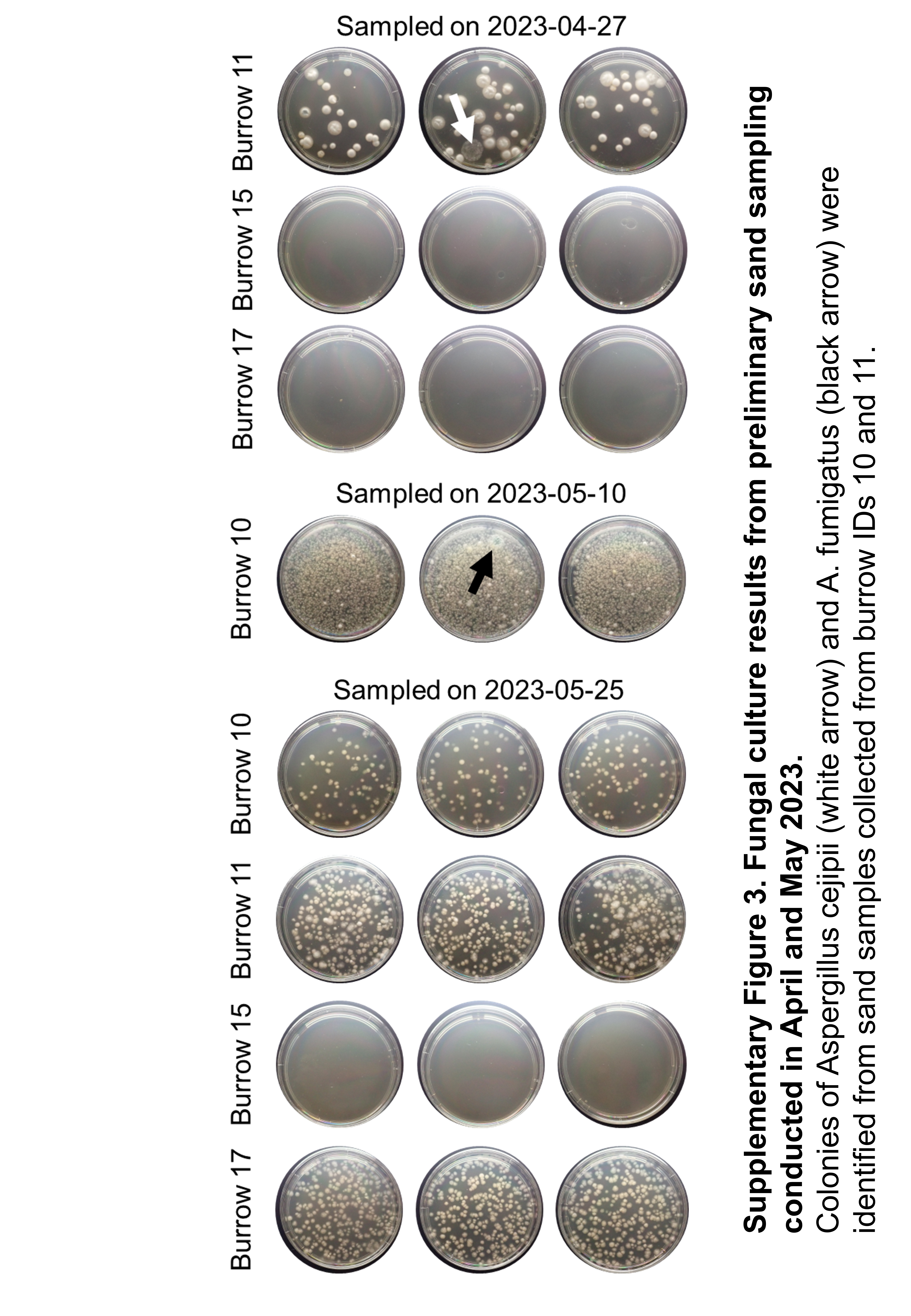

Supplement: Supplementary file 4 [file Image_3.TIF]

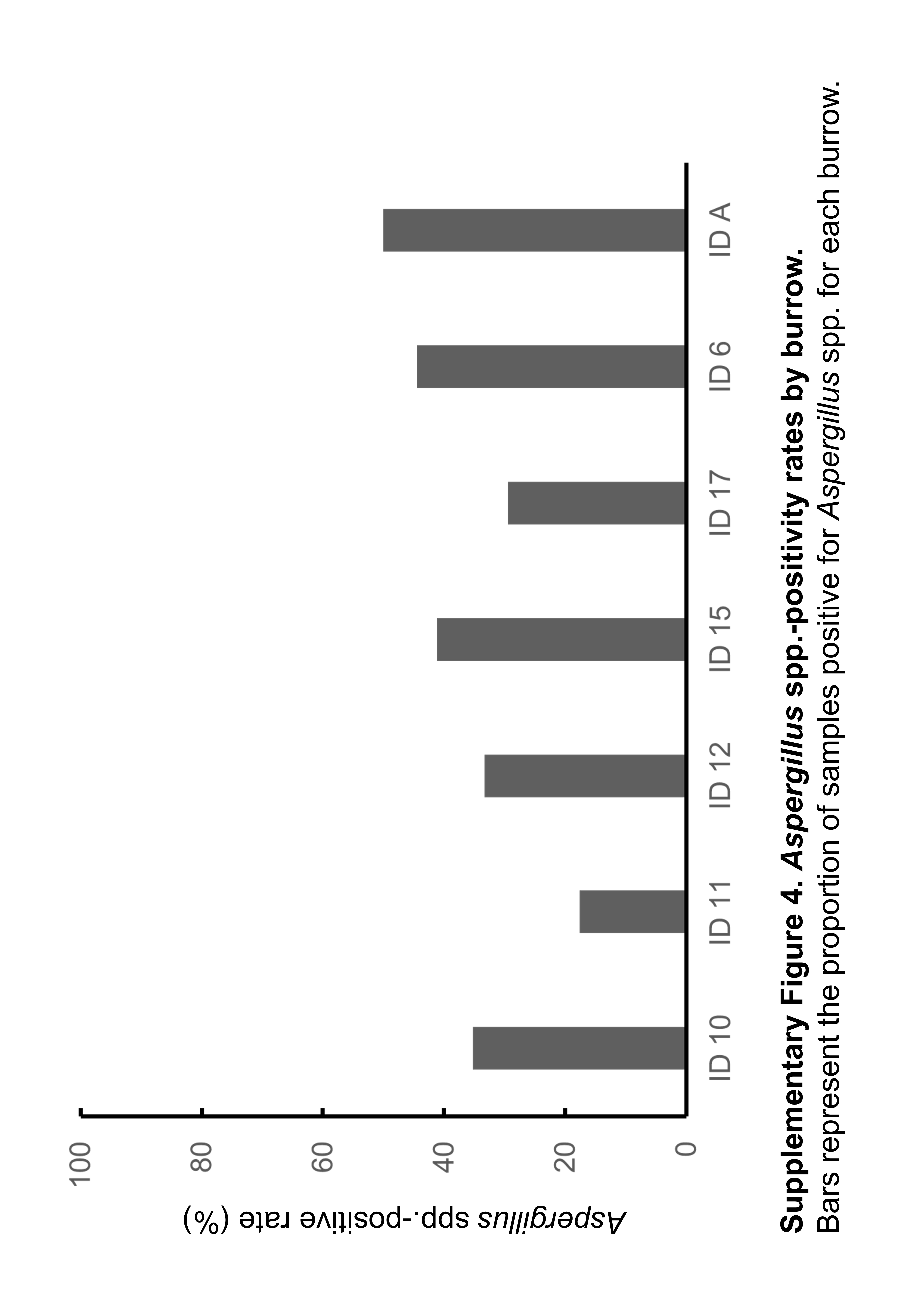

Supplement: Supplementary file 5 [file Image_4.TIF]
