# Supplementary material for: Seasonal variation in Aspergillus abundance in captive penguin burrow sands and its implication for aspergillosis risk in Japan
Source: Front Vet Sci. 2026 Jan 13;12:1708049. doi: 10.3389/fvets.2025.1708049 (PMC12836384; doi:10.3389/fvets.2025.1708049)
Supplement: Supplementary file 6 [file Table_1.DOCX]

| Supplementary Table 1. Demographic information of the zoo penguin colony used in this study. | | | | | | |
| --- | --- | --- | --- | --- | --- | --- |
| Date (YYYY/MM/DD) | | Population | | | Age range  (years) | Aspergillosis cases^†1^ |
| From | To | Total | Male | Female |  |  |
| 2023/04/01 | 2023/04/04 | 47 | 23 | 24 | 3 – 23 |  |
| 2023/04/05 | 2023/04/13 | 45 | 22 | 23 | 3 – 23 |  |
| 2023/04/14 | 2023/06/06 | 43 | 21 | 22 | 3 – 23 |  |
| 2023/06/07 | 2023/07/24 | 42 | 21 | 21 | 3 – 24 | a |
| 2023/07/25 | 2023/08/31 | 40 | 20 | 20 | 3 – 24 | a |
| 2023/09/01 | 2023/09/07 | 40 | 20 | 20 | 3 – 24 | a |
| 2023/09/08 | 2023/09/19 | 40 | 20 | 20 | 3 – 24 | a |
| 2023/09/20 | 2023/09/21 | 38 | 19 | 19 | 4 – 24 | a |
| 2023/09/22 | 2023/09/29 | 40 | 20 | 20 | 4 – 24 | a |
| 2023/09/30 | 2023/10/09 | 38 | 19 | 19 | 4 – 24 | a, b |
| 2023/10/10 | 2023/10/22 | 36 | 18 | 18 | 4 – 24 | a, b |
| 2023/10/23 | 2023/10/23 | 34 | 17 | 17 | 5 – 24 | a, b |
| 2023/10/24 | 2023/10/24 | 29 | 14 | 15 | 5 – 24 | a, b |
| 2023/10/25 | 2023/11/26 | 28 | 14 | 14 | 5 – 24 | a, b |
| 2023/11/27 | 2024/03/12 | 32 | 16 | 16 | 5 – 24 | a, b |
| 2024/03/13 | 2024/03/18 | 30 | 15 | 15 | 6 – 24 | b |
| 2024/03/19 | 2024/04/14 | 28 | 14 | 14 | 6 – 24 | b |
| 2024/04/15 | 2024/04/22 | 33 | 18 | 15 | 1 – 24 | b |
| 2024/04/23 | 2024/05/14 | 38 | 19 | 19 | 1 – 24 | b |
| 2024/05/15 | 2024/05/15 | 45 | 21 | 24 | 1 – 24 | b |
| 2024/05/16 | 2024/08/31 | 50 | 21 | 29 | 1 – 25 | b, c^†2^ |
| 2024/09/01 | 2024/10/31 | 50 | 21 | 29 | 1 – 25 | c^†2^ |
| ^†1^ Consistent alphabetic labeling indicates continuity of treatment for each identical individual.  ^†2^ Because the individual was in a different location at the time of initial onset, it is considered that the infection did not occur within the facility. | | | | | | |
